# Non-progressive Nonimmune Hydrops Fetalis Caused by a Novel Mutation in *GUSB* Gene

**Published:** 2020

**Authors:** Asieh MOSALLANEJAD, Mohammadreza ALAEI, Saeed Reza GHAFFARI, Maryam RAFATI, Hedyeh SANEIFARD

**Affiliations:** 1 Department of Pediatric Endocrinology and Metabolic diseases, Mofid children hospital, Shahid Beheshti University of Medical Sciences, Tehran, Iran; 2 Reproductive Biotechnology Research Center, Avicenna Research Institute, Shahid Beheshti University of Medical Sciences, Tehran, Iran

**Keywords:** Mucopolysaccharidosis, Sly Syndrome, GUSB Gene, Hydrops Fetalis

## Abstract

**Objectives: **Mucopolysaccharidosis type VII (MPS VII) or Sly syndrome is a rare autosomal recessive disorder caused by deficiency of β-glucuronidase enzyme, which is involved in degradation of glycosaminoglycans. The lack of β-glucuronidase in this lysosomal storage disorder is characterized by various manifestations such as nonimmune hydrops fetalis, spinal deformity, organomegaly, dysostosis multiplex, intellectual disability, and eye involvement. It is caused by a mutation in *GUSB* gene located on chromosome 7 q11. The current study reported an Iranian female with MPS VII and a novel mutation (c.542G>T, p.Arg181Leu) in *GUSB* gene.

## Introduction

Sly syndrome or mucopolysaccharidosis type VII (MPS VII) (OMIM 253220) is a rare genetic disorder inherited in an autosomal recessive mode. The condition was first described by Sly et al. (1). The GUSB gene mutation results in β-glucuronidase enzyme deficiency and consequently, accumulation of glycosaminoglycans (GAGs) in lysosomes (2).The accumulation of heparin sulfate, dermatan sulfate, chondroitin-4 sulfate, and chondroitin-6 sulfate causes various manifestations in different organs (3). The patients express a wide range of phenotypic heterogeneity, probably due to overexpression or multiallelic mutations (4). The most commonly observed characteristic is nonimmune hydrops fetalis at birth or prenatal period. Other main features of this disorder are corneal clouding, heavy eyebrows, coarse facies, dysostosis multiplex, macroglossia, respiratory infection, sensorineural hearing loss, kyphosis, and hepatosplenomegaly (5). Nonimmune hydrops fetalis is a prominent symptom before birth.

In conclusion, lysosomal storage disease, especially MPS VII, should be considered in the case of nonimmune hydrops fetalis.

The current study reported an Iranian female presented with non-progressive nonimmune hydrops fetalis and a novel mutation (c. 542G>T, p.Arg181Leu) in GUSB gene.

## Case Report

A 28-month-old female patient was referred to Endocrine Clinic due to kyphosis and hepatosplenomegaly. She was the first child of healthy consanguineous Iranian parents without any diseases and a history of abortion in her mother, but a history of two stillbirths was reported in her uncle’s family due to hydrops fetalis. The patient had received intrauterine transfusion of packed cell at 23rd and 28th week of gestation due to hydrops fetalis. She was delivered spontaneously at 37th week of gestation. There was no symptom of hydrops fetalis or ascites at birth. She was hospitalized three times due to pneumonia. On physical examination, she had short stature with a coarse facies, thoracolumbar kyphosis, and scoliosis; hepatosplenomegaly and umbilical hernia were also present. Developmental and neurologic examinations revealed significant delay of fine motor skills and speech. She had no seizure and head circumference was normal. X-ray examination showed signs of dysostosis multiplex, scoliosis, kyphosis, and vertebral beaking.

According to these findings, urine was tested for GAGs and the result was positive. Venous blood sample was also collected and used for DNA extraction and whole exome sequencing was carried out using Ion Torrent platform (Life Technologies Corporation, Carlsbad, CA). Library and template preparation followed by high throughput sequencing were performed based on the manufacturers’ guidelines. Variants were called, filtered, and analyzed using the Ion Reporter pipeline (Life Technologies Corporation ,USA), focusing on genes involved in hydrops fetalis. The candidate variant was confirmed through Sanger sequencing along with co-segregation analysis. Whole exome sequencing revealed a DNA variant (c.542G>T, p.Arg181Leu, NM_001284290.1) in *GUSB* gene, in the affected individual. DNA variants are shown in Table 1.

**Table 1:** The Result of Genetic Study of the Patient (Whole Exome Sequencing)

Gene	Chromosomal Location	NM-No	Variant Location	Detected Variant	Genotype	Sanger Verification
GUSB	Chr7:65439991	NM_001284290.1	Ex4	c.542G>T (p.Arg181Leu)	Homo	Confirmed

The result of further molecular genetic studies of the other family members are shown below: 

**Table 2:** The Result of Further Molecular Genetic Studies of the Other Family Members

_Individual ID_	_GUSB_ (c. 542G>T,_ p.Arg181Leu)_
_3:3 _ _(Proband)_	_Homozygous_
_2:3 _ _(Father)_	_Heterozygous_
_3:8 _ _(Mother)_	_Heterozygous_
_ 3:2_	_Heterozygous_
_3:7_	_Heterozygous_

**Figure:**
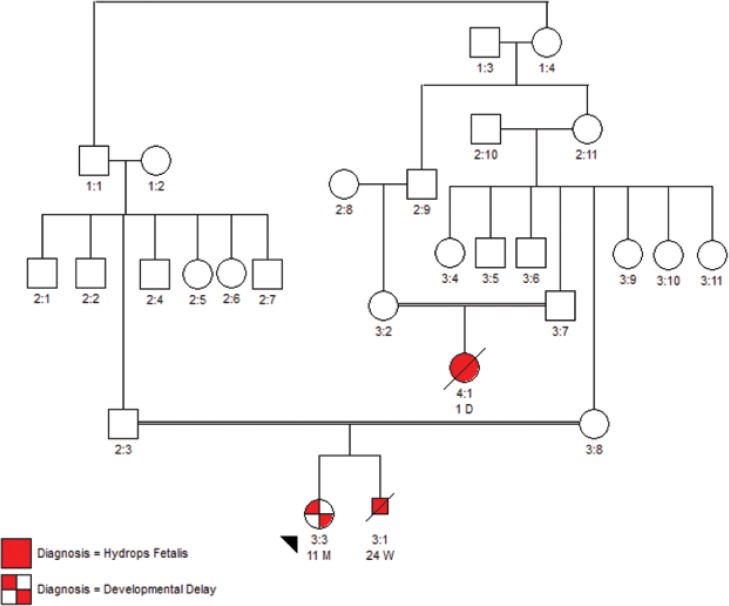


## Discussion

Sly syndrome or MPS VII is a potential lethal disease caused by mutations in the *GUSB* gene (6). These mutations cause glucuronidase enzymes deficiency and GAG accumulation in lysosomes. The exact incidence of this disease is not known, but it is estimated to be 1: 300,000 to 1: 2,000,000 (7). A number of patients may not be diagnosed due to prenatal deaths (5). Patients with MPS VII show a wide range of symptoms in terms of the phenotype. The most prominent symptom before birth is the presence of nonimmune hydrops fetalis. Up to 15% of the causes of hydrops fetalis are due to lysosomal storage diseases (8). In some studies, it is reported that the presence of hydrops fetalis is associated with severe fetal disease and postnatal death (9). However, in the study by Montanto et al., performed on 56 patients with Sly syndrome, 13 patients with a history of nonimmune hydrops fetalis in embryonic period showed a wide range of symptoms after birth; therefore, it seems that the presence of hydrops fetalis is not a reliable predictive factor to determine the severity of the disease (5). The current study patient, despite the presence of hydrops fetalis and receiving two intrauterine packed cells during pregnancy, survived for 2.5 years. A wide range of heterogeneity phenotypes is observed in the MPS VII (2,5,8). The mutation in the *GUSB* gene affects the structure, function, and stability of the *GUSB* gene and the glucuronidase enzyme (6). The accumulation of GAGs in lysosomes increases the size of lysosomes, and as a result causes oversized organs. On the other hand, GAGs interact with the function of other proteins in lysosomes and affect other cell functions (7). It seems that the involvement of some organs in the disease is due to these interactions. Monatano et al., examined the clinical symptoms in 56 patients with MPS VII. In their study, the most common symptom was coarse facial features, observed in 87% of patients. Other progressive symptoms included corneal clouding, enlarged tongue, heart abnormalities, organomegaly, neurodevelopmental delay, and skeletal deformity (5). The current study patient showed these signs on admission with a slow progression over two years of follow-up.The presentation of the under study patient was a nonimmune hydrops fetalis. According to the study by Montano, approximately 40% of patients with nonimmune hydrops fetalis show a wide range of outcomes. The hydrops fetalis may be caused by hepatic sinusoidal infiltration and obstruction that result in a generalized edema (5). Cardiovascular involvement may also play a role in the development of the edema (10). Therefore, lysosomal storage disease, especially MPS VII, should be considered in the presence of nonimmune hydrops fetalis.Determination of the level of urinary GAG helps to evaluate patients suspected of Sly syndrome (11). In some studies, a low level of GAG secretion in urine was associated with longer survival (8), but some other studies stated that urinary GAG level was not an appropriate and reliable indicator to predict the severity of the disease (7). This difference seems to be due to the method used to study the urine GAG. In the current study patient, the result of the GAG quality assay was positive, but the quantity assessment was not performed. The genotype-phenotype correlation studies are difficult due to the rare nature of this disease (7). Conzelmann et al., described the theory of residual enzyme activity to explain the differences in severity of the disease (12). Based on their theory, various mutations in the *GUSB* gene cause different degrees of reduced activity or deficiency of the enzyme and this difference leads to different clinical symptoms and variations in severity of the disease (12).In the current study patient, a genetic study was conducted indicating a novel mutation in the *GUSB* gene. This variant (c.542G>T, p.Arg181Leu, NM_001284290.1) was not previously reported; therefore, Sanger sequencing was performed and the parents of the patient, as well as two other members of the family, were examined for this variant. The obtained results indicated that the family members were heterozygotes for this variant. The variant was classified as a “variant of unknown clinical significance (VUS) based on the American College of Medical Genetics and Genomics guidelines. There is a need to study the enzyme in order to prove a new mutation. However, since the result of the enzyme assessment was not available, this variant was classified as a VUS.

**In Conclusion**

lysosomal storage disease, especially MPS VII, should be considered in the case of nonimmune hydrops fetalis.
